# Carbon dioxide de-airing in minimal invasive cardiac surgery, a new effective device

**DOI:** 10.1186/s13019-018-0824-4

**Published:** 2019-01-17

**Authors:** Jesper Nyman, Peter Svenarud, Jan van der Linden

**Affiliations:** 1 Division of Perioperative Medicine and Intensive Care, Section Cardiothoracic Surgery and Anesthesiology, Karolinska University Hospital Solna, SE-17176 Stockholm, Sweden; 2 Department of Molecular Medicine and Surgery, Karolinska Institutet, Stockholm, Sweden; 3 Section of Cardiac Surgery, Karolinska University Hospital, Stockholm, Sweden

**Keywords:** Carbon dioxide, Minimal invasive cardiac surgery, de-airing, Emboli

## Abstract

**Background:**

Arterial air embolism during open heart surgery may cause postoperative complications including cerebral injury, myocardial dysfunction, and dysrhythmias. Despite standard de-airing techniques during surgery large amounts of arterial air emboli may still occur, especially during weaning from cardiopulmonary bypass. To prevent this insufflation of carbon dioxide in the wound cavity has been used since the 1950s. The aim of this study was to assess a new mini-diffuser for efficient carbon dioxide de-airing of a minimal invasive cardiothoracic wound cavity model. Up until now no device has been evaluated for this purpose.

**Methods:**

A new insufflation device, a mini-diffuser, was tested. A thin plastic tube was used as control. The end of the mini-diffuser or the control, respectively, was positioned in a minimal invasive thoracic wound model. Remaining air content was measured during steady state and during intermittent suction with a rough suction device at different carbon dioxide flow rates. Measurements were also carried out in the open surgical wound during minimal invasive aortic surgery in six patients.

**Results:**

**T**he air content was below 1% 4 cm below the surface of the open wound model during continuous carbon dioxide inflow of 2–10 L/min with the mini diffuser. In comparison, carbon dioxide insufflation via the open-ended tube resulted in a mean air content between 10 and 75%. The mean air content of the wound model remained below 1% at a carbon dioxide flow rate of 3–5 L/min during intermittent application of a suction device with a suction rate of 15 L/min. In 6 patients undergoing minimal invasive aortic valve replacement air content in the open surgical wound remained below 1% at a continuous carbon dioxide flow rate of 5 and 8 L/min via the mini-diffuser, respectively.

**Conclusions:**

The mini diffuser was effective for carbon dioxide de-airing, i.e. < 1% remaining air, of a minimal invasive cardiothoracic wound cavity model with and without intermittent rough suction as well as in patients undergoing minimal invasive aortic valve surgery.

## Background

Arterial air embolism during open heart surgery may cause postoperative complications including cerebral injury, myocardial dysfunction, and dysrhythmias [1–7]. Despite standard de-airing techniques during surgery large amounts of arterial air emboli may still occur, especially during weaning from cardiopulmonary bypass [8, 9]. To prevent this, insufflation of carbon dioxide (CO_2_) in the wound cavity has been used since the 1950s. As CO_2_ is > 25 times [10] more soluble than air in blood at 37–38 °C, arterial CO_2_-emboli are much better tolerated than air emboli [2–5, 11, 12]. Furthermore, CO_2_ has a 50% higher density than air and will hence displace air by gravity in the open wound cavity.

The use of CO_2_ in minimal invasive cardiac surgery is probably more important compared with open cardiac surgery, as minimal invasive cardiac surgery does not permit normal de-airing maneuvers. Furthermore, the minimal invasive surgical wound cavity is much smaller, which necessitates a much smaller administration device for CO_2_ delivery. So far no device has been evaluated for this purpose.

The aim of this study was to assess a new device, a mini-diffuser, for efficient CO_2_ de-airing of a minimal invasive cardiothoracic wound cavity model and in patients undergoing minimal invasive aortic valve surgery.

## Materials and methods

### Insufflation devices and experimental wound model

**Fig. 1:**
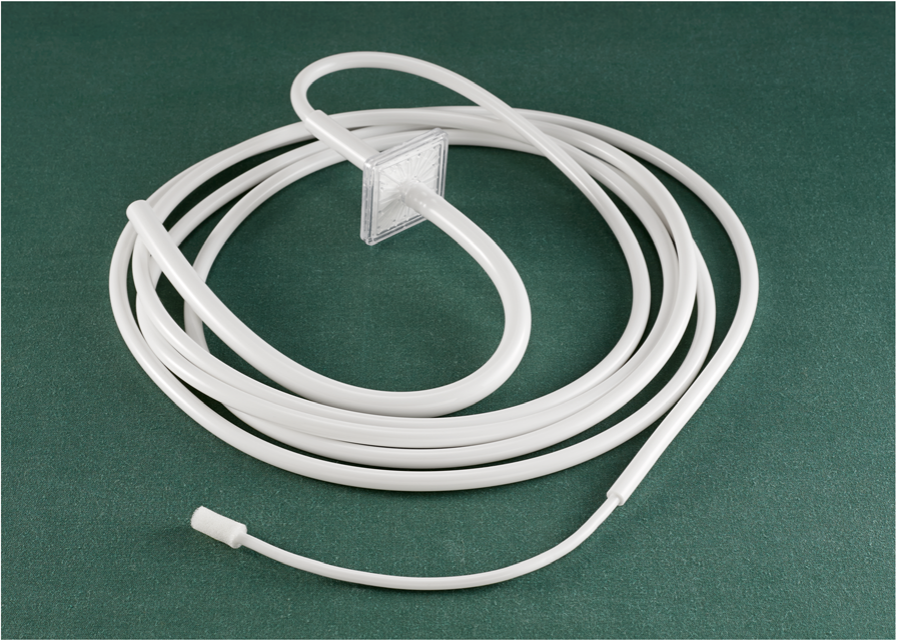
Photo of the mini-diffuser

A disposable insufflation device, the CO_2_-diffuser (CarbonMini® Cardia Innovation AB, Stockholm, Sweden) consists of a 2 m long PVC tube with inner diameter ¼ inch (6.35 mm), a bacterial filter (ORO1H from Pall Medical, Portsmouth, U.K.), a 0.20 m PVC tube with inner diameter 1 mm inside which a metal thread runs, and a soft polyurethane cylindrical diffuser with a diameter of 7 mm and a length of 17 mm, giving a surface area of 374 mm^2^ **(**Fig. 1**)**. A plastic tube with an inner diameter of 1 mm was used as control, as this is a frequently used device to insufflate CO_2_ in wound cavities in minimal invasive cardiac surgery.

The size of the minimal invasive cardiothoracic wound model was based on measurements (median) of the minimal invasive cardiothoracic wound cavities of three patients undergoing minimal invasive cardiac surgery through a partial sternotomy. The opening of the wound model was 70 mm in diameter and it had a depth of 44 mm. Furthermore, a simplified aortic model with a size of 50x40x40 mm was positioned at its base.

### Instrumentation

The CO_2_ gas flow was measured with a back-pressure compensated O_2_-flowmeter, as CO_2_-flowmeters are rarely used in clinical practice. The O_2_-reading scale was adjusted for CO_2_ by a universal flowmeter (ABB/Fisher & Porter, Göttingen, Germany), because of the higher density of CO_2_ gas. The universal flowmeter consisted of a measuring tube (FP ¼-16 G-5/81) with a spherical stainless steel float (SS-14). However, the universal flowmeter was not used for direct measurements in the study, due to its lack of compensation for back-pressure. This problem was avoided during the calibration by measuring the CO_2_ outflow distal to the end of the insufflation devices. The tube reading scale of the universal flowmeter was calculated for the used gas (medical CO_2_, AGA Gas AB, Stockholm, Sweden) at 20 degrees C and at 1013 mbar with a computer program (FlowSelect version 2.0, ABB/Fisher & Porter, Göttingen, Germany). Coronary suction was not used at the time of measurement. The suction effect of the rough suction was 5, 10, and 15 L/min, controlled by a flowmeter.

The air displacement was analyzed by assessing the air content inside the model. The air content was measured by continuous sampling of the oxygen (O_2_) content given by the formula:

$$ air\%=\frac{0_2\%}{0_2(ref.)\%}.100 $$

where O_2_ (ref.) % = 21% (14) which is the normal O_2_-content in atmospheric air at sea-level. The O_2_-content was measured with an O_2_-sensor (CheckMate 9900, PBI Dansensor, Denmark). This O_2_-instrument has a sampling volume of < 2 ml, a response time of < 2 s, a range of measurement of 0.0001 to 100% O_2_, and an accuracy of 1% of the measured value (the response time of optical infrared CO_2_-sensors are usually > 10 s with a fix accuracy of approximately 2% CO_2_-units in the range 0–100%). The sampling probe was 2.0 mm thick sterile and disposable pressure monitoring tube (B. Braun, Meisungen, Germany).

### Measurements

The tip of the insufflation device was positioned towards the bottom of the experimental wound cavity at a depth of 25 mm from the wound surface. CO_2_ was supplied to the wound cavity via the device at a flow of 2, 3, 4, 5, and 10 liters per minute (L/min). The air content was measured during steady state in each quadrant of the wound cavity 15 mm below the wound area opening and at the base of the wound cavity.

The air displacement efficiency of the new mini-diffuser and the control device for insufflation was assessed during static conditions without suction. The efficiency of the mini-diffuser was thereafter further explored with the addition of the varying degrees of suction, applied via a suction device (Kendall Argyle Yankauer suction tube (fine), Tyco Healthcare, 4.0 mm, Ireland) either for 2 s, intermittent suction, or for continuous suction at a depth of 15 mm from the wound area opening. The O_2_-content was measured at a depth of 40 mm at steady state, 60 s of stable values, before start of intermittent (2 s) or continuous suction, with 10 measurements during each condition. After start of intermittent suction the O_2_-content was measured every 5 s for 25 s. After start of continuous suction the O_2_-content was measured every 10 s for 90 s. The remaining CO_2_ in the model was removed with the help of a rough suction device before every change of CO_2_ flow rate, insufflation device, or measuring position.

In six patients undergoing minimal invasive aortic valve surgery through a 6 cm long J-shaped partial sternotomy from the jugulum to the third intercostal space, air content was measured 4 cm below the wound surface when insufflations the open wound with a mini diffuser, positioned contra-lateral to the measuring site, at a CO_2_ flow rate of 5 and 8 L/min, respectively.

### Statistics

Data are presented as mean ± SD and were analyzed with a commercially available statistic program (SPSS, version 19, IBM). Due to the small numbers, we chose to use nonparametric tests, the Mann-Whitney U-test and the Kruskall-Wallis test, which were applied when appropriate. A *p*-value less than 0.05 was considered significant.

## Results

**Fig. 2:**
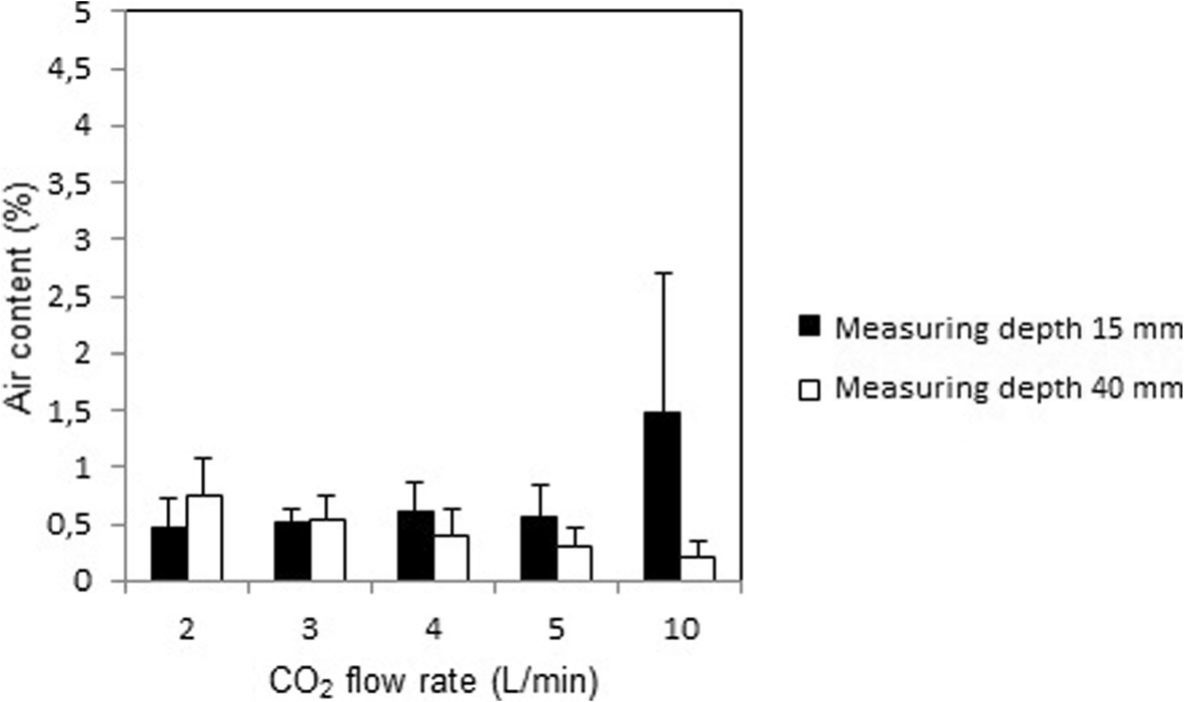
Measured air content at steady state when insufflating CO_2_ at various flow rates into a minimal invasive cardiothoracic wound cavity model with a new CO_2_ insufflation device. Air content was measured at a depth of 15 mm and 40 mm from the opening surface of the wound cavity model

With the mini-diffuser the air content at 15 mm depth in the wound model was 0.5 ± 0.2%, 0.5 ± 0.2%, 0.6 ± 0.3%, 0.6 ± 0.3%, and 1.5 ± 1.2%, respectively, during a continuous CO_2_ inflow of 2, 3, 4, 5, and 10 L/min, respectively. Forty mm below the surface the corresponding values were 0.8 ± 0.3%, 0.5 ± 0.2%, 0.4 ± 0.2%, 0.3 ± 0.2%, and 0.2 ± 0.1%, respectively (Fig. 2).

**Fig. 3:**
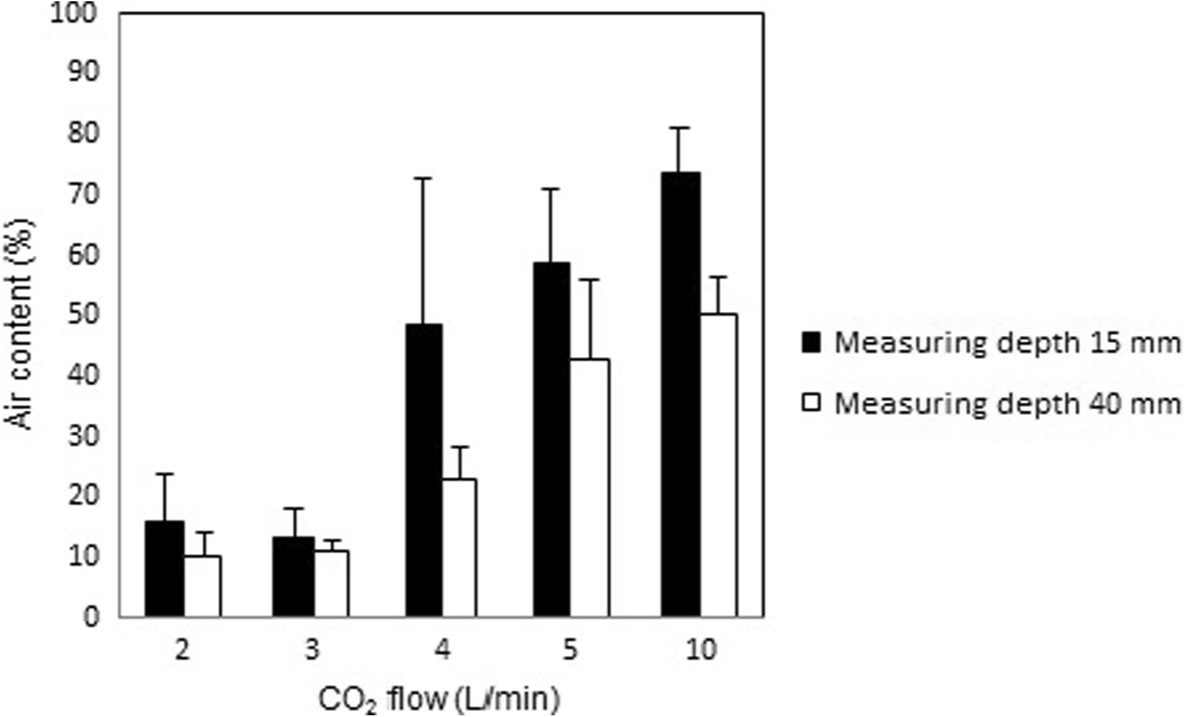
Measured air content at steady state when insufflating CO_2_ at various flow rates into a minimal invasive cardiothoracic wound cavity model with an open tube (inner diameter of 1 mm). Air content was measured at a depth of 15 mm and 40 mm from the opening surface of the wound cavity model

When the control device, the open-ended tube, was used to insufflate the wound cavity model, the mean air content was between 10 and 20% at a CO_2_ flow of 2 and 3 L/min, and the mean air content increased to between 20 and 75% at flow rates of 4, 5 and 10 L/min (Fig. 3). Thus, the mini-diffuser device de-aired the wound cavity model much more efficiently at all measured flows (*p* = 0.02) when compared with the control.

**Fig. 4:**
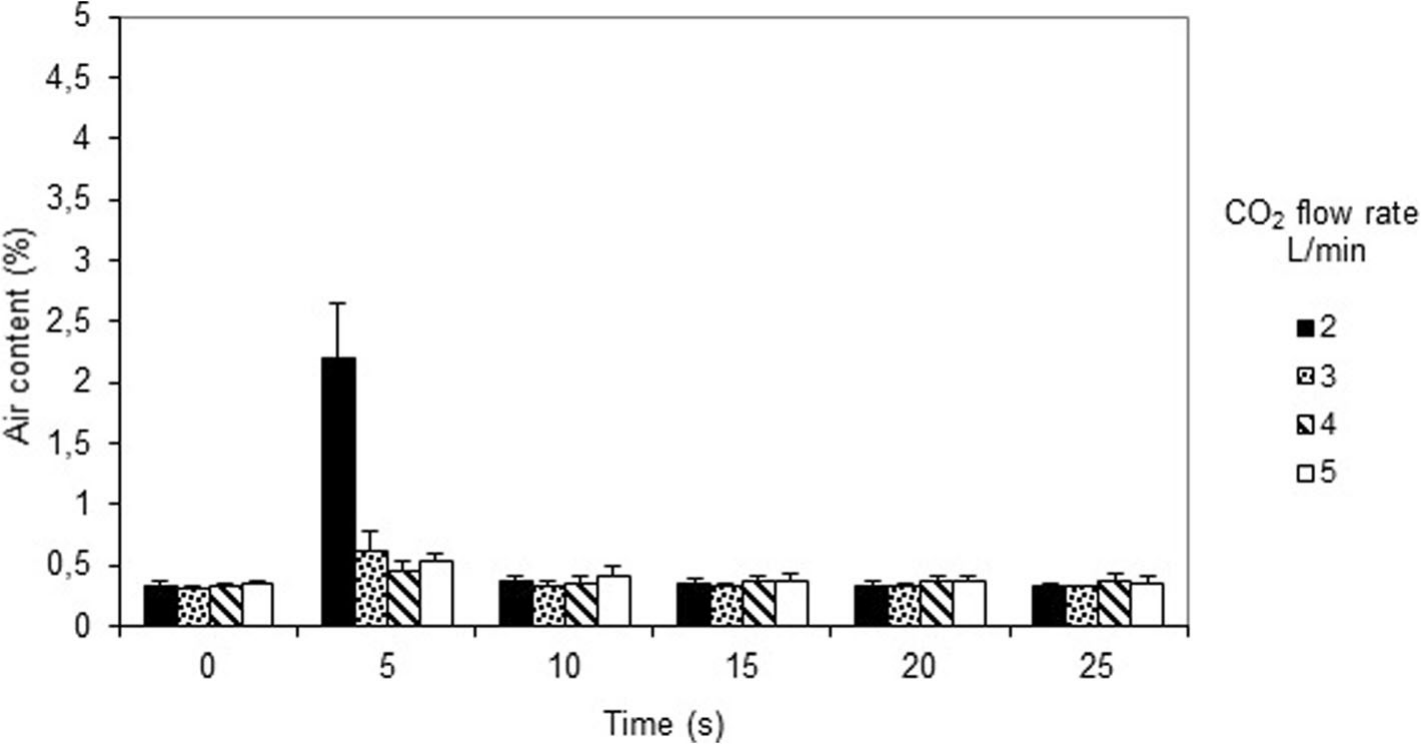
Air content measured every five seconds, when using the new CO_2_ insufflation device and intermittent rough suction rate of 10 L/min for two seconds from time zero. CO_2_ was insufflated at a flow rate of 2, 3, 4, and 5 L/min, respectively

Figure 4 depicts the air content when an intermittent rough suction force of 10 L/min was applied for two seconds. A very small but significant rise in air content occurred after 5 s for all flows (*p* < 0.001), after which the air content reversed to steady state levels after 10 s for all flows. Thus, despite a two second intermittent rough suction force of 10 L/min the air content remained below 1% at all CO_2_ flow rates, except when the flow was only 2 L/min, which resulted in an increase of the air content to approximately 2.4% (*p* < 0.001).

**Fig. 5:**
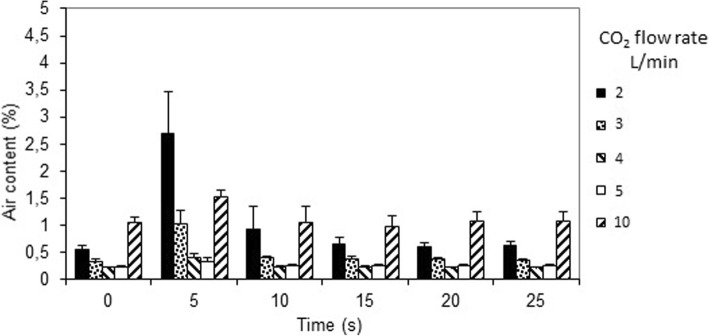
Air content measured every five seconds, when using the new CO_2_ insufflation device and intermittent rough suction rate of 15 L/min for two seconds from time zero. CO_2_ was insufflated at a flow rate of 2, 3, 4, 5, and 10 L/min, respectively

Figure 5 illustrates the air content when an intermittent rough suction force of 15 L/min was applied for two seconds. Similarly to what was seen with the addition of a suction rate of 10 L/min, the mean air content in the model rose significantly after 5 s at all CO_2_ flow rates (*p* < 0.001). However, the mean air content only increased above 1% at a CO_2_ flow rate of 2 and 10 L/min. The air content returned to steady state values for all CO_2_ flow rates already after 10 s, which was similar to the pattern seen when applying a suction rate of 10 L/min.

**Fig. 6:**
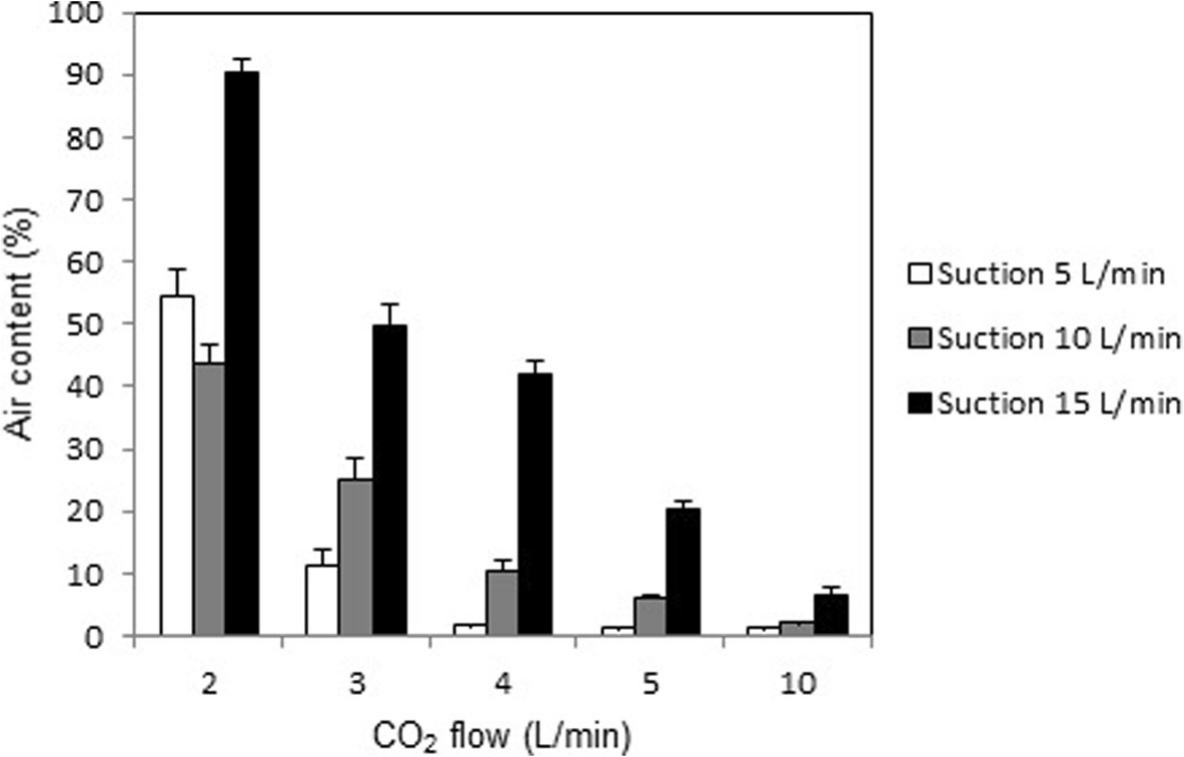
Measured air content at steady state when using the new CO_2_ insufflation device and continuous rough suction force of 5, 10, and 15 L/min. CO_2_ was insufflated at a flow rate of 2, 3, 4, 5, and 10 L/min, respectively

The application of a continuous rough suction rate of 5, 10, and 15 L/min, at CO_2_ flow rates of 2, 3, 4, 5, and 10 L/min, respectively, resulted in a clear pattern. With every increment in CO_2_ flow rate the air content decreased significantly (p < 0.001) for all three suction rates, except that the air content remained statistically unchanged at a suction rate of 5 L/min when the flow rate was increased from 5 to 10 L/min (*p* = 0.21). The mean air content was ≤10% at a CO_2_ flow rate 4, 5, and 10 L/min, respectively, when applying a continuous rough suction rate of either 5 or 10 L/min (Fig. 6). With a CO_2_ flow rate of 10 L/min the air content remained below 10% for a continuous suction rate of 5, 10, and 15 L/min.

The mean air content in the open surgical wound of the 6 patients undergoing minimal invasive aortic valve replacement was 0.4 ± 0.5% and 0.6 ± 0.7% at a continuous CO_2_ flow rate of 5 and 8 L/min via the mini-diffuser, respectively. None of the patients experienced any signs of postoperative deterioration of cerebral or cardiac function **(**Table 1**)**.

**Table 1:** Table summarizing results of the new device for 6 adult patients undergoing minimal invasive aortic valve replacement

	Mini-diffuser		Postoperative stroke or cognitive dysfunction	Decrease in regional right or left ventricular function after CPB	Ventricular arrhythmias after CPB
CO_2_ flow rate	5 L/min	8 L/min	0/6	0/6	0/6
Mean air content % in surgical cavity	< 1%	< 1%			

*CPB* Cardio-pulmonary bypass

## Discussion

Since many years CO_2_ is insufflated into the open surgical wound during conventional open cardiac surgery to prevent air embolism. However, studies about a decade ago showed that a conventional administration of CO_2_ with an open-ended tube caused a high velocity jet into the open surgical wound that created turbulence and thus poor de-airing [13]. Consequently, efficient de-airing could only be achieved by using a device with a diffuser at its end that enables a high gas flow but with a low velocity output [13]. This device, which is used in open cardiac surgery in most countries in the western world (based on information from the manufacturer, Cardia Innovation AB, Stockholm, Sweden), is however by many surgeons considered too large to be utilized in minimal invasive surgery. Earlier studies have also shown that efficient de-airing can only be accomplished by positioning the CO_2_ insufflation device inside the wound cavity and not next to its orifice [13]. Thus, there is a need for a new device that is small enough not to interfere with surgery and at the same time is large enough to enable efficient de-airing of a small open surgical wound. The new device was designed to meet these conditions and was in this study tested for its efficiency in comparison with an open-ended tube that is alternatively used for this purpose in minimal invasive cardiac surgery.

The mini-diffuser was evaluated in an experimental setup to enable us to vary several basic variables known to influence de-airing during surgery in an open wound, including geometry, flow rates, and a suction force from a suction device positioned in the wound cavity [14]. The size of the surgical wound model was based on measurements from the size of the partial sternotomy wound in adult patients undergoing minimal invasive aortic valve surgery. Efficient CO_2_ de-airing also presupposed continuous insufflation of the CO_2_ gas to prevent air from entering the open heart and vessels, since otherwise air trapped inside the heart and vessels cannot be removed. The CO_2_ flow rate was thus varied between a low rate of 2 L/min, which should be able to counteract against the strong force of diffusion from the ambient air, and a very high flow rate of 10 L/min, which is the recommended flow rate when applying a large diffuser device in adults undergoing open cardiac surgery via a complete sternotomy. Furthermore, the air content was measured not only at the base of the model but also close to its opening area, where the highest part of the ascending aorta is situated and where the diffusion towards ambient air will have its highest impact. Another factor that may influence the efficiency of de-airing is the intermittent use of suction devices during surgery. Thus, we positioned a surgical rough suction device inside the wound cavity model and applied a suction force of 5, 10, or 15 L/min at the site of the artificial ascending aorta where a bleeding could be expected to occur.

Static de-airing with the open-ended tube resulted in a remaining air content that was above 10% at 2 L/min and increased substantially with each increment in CO_2_ flow rate. These results can be explained by the turbulence caused by the high outflow velocity from the small orifice of the open-ended tube. In contrast, static de-airing of the minimal invasive wound cavity model with the new mini diffuser resulted in an air content that remained below 1% at CO_2_ flow rates between 2 and 5 L/min, and below 2% at a CO_2_ flow rate of 10 L/min (Fig. 2). With the new mini-diffuser, the flow rates of 3–5 L/min has the advantage of minimizing the remaining air content, whereas the highest tested flow rate of 10 L/min will minimize the dynamic influence of surgical maneuvers and suction devices on de-airing.

Two types of suction devices are usually used in cardiac surgery. A low force suction device is often applied at the base of the surgical wound cavity for continuous drainage of blood at a suction rate of 0.25 to 1 L/min, which will only interfere with de-airing if it is close to the CO_2_ insufflation rate. In contrast, rough suction devices with a high suction rate used intermittently for evacuation of local blood from the operating area enable optimal surgical exposure and visualization without interfering with CO_2_ de-airing during conventional cardiac surgery with complete sternotomy [14].

When a rough suction force of 10 L/min was applied intermittently, the measured air content in the wound cavity model was kept below 1% at CO_2_ flows between 3 and 5 L/min, whereas the air content rose above 2.5% at a CO_2_ flow rate of 2 L/min. A further increase of an intermittent suction force to 15 L/min would necessitate a higher insufflation rate of CO_2_. Indeed, when applying an intermittent suction rate of 15 L/min, the air content was kept at < 1% with CO_2_ flow rates of 4 to 5 L/min, and slightly above 1% at a CO_2_ flow rate of 10 L/min.

In comparison, when a rough suction rate of 15 L/min was applied continuously in the wound cavity model at CO_2_ flow rates of 2 to 5 l, the air content was above 20%. With a CO_2_ flow rate of 10 L/min the air content decreased to < 7%. Accordingly, when using rough suction force of 10 L/min continuously in the minimal invasive wound cavity model, the air content was > 20% with a CO_2_ flow rate of 2 and 3 L/min, whereas with a with a CO_2_ flow rate of 4, 5, and 10 L/min the air content was ≤10%. However, to avoid air trapping the use of continuous suction should be avoided, especially during cannulation and opening of the large vessels and heart chambers. The use of continuous suction in a situation of profuse bleeding will usually be performed with coronary suction devise at a much lower suction force, and will thus not affect de-airing.

The new mini diffuser was then tested clinically during minimal invasive aortic surgery and was found to de-air the open surgical wound cavity efficiently, i.e. less than 1% remaining air, at a continuous flow rate of 5 and 8 L/min, respectively. Thus, one could use the higher flow rate of 8 L/min if one expects to use a rough suction device frequently or for longer durations.

There are some limitations to the study that should be acknowledged. We performed most of the measurements using a model instead of testing the new device at different measuring points, flow and suction rates in a clinical setting. However, all these measurements would have been ethically questionable to perform in patients. Furthermore, we did not perform an evaluation of the possible impact on cognitive function by prevention of air embolism via continuous CO_2_ de-airing during minimal invasive aortic valve surgery. On the other hand, prevention of air embolism should be possible by efficient CO_2_ de-airing of the open surgical wound, and thus should prevent air from entering the open heart and great vessels.

In earlier clinical trial in patients undergoing open heart surgery through a full sternotomy [15] we randomized patients to insufflation of the open cardiothoracic cavity with CO_2_ via a standard gas diffuser, or not. Microemboli were ascertained by intraoperative transoesophageal echocardiography (TOE) and recorded on videotape from the moment that the aortic cross-clamp was released until 20 min after end of cardiopulmonary bypass (CPB). The surgeon performed standard de-airing manoeuvres without being aware of TOE findings. Postoperatively, a blinded assessor determined the maximal number of gas emboli during each consecutive minute in the left atrium, left ventricle, and ascending aorta. The median number of microemboli registered during the whole study period was 161 in the CO_2_ group versus 723 in the control group (*P* < 0.001). Corresponding numbers for the left atrium were 69 versus 340 (P < 0.001), left ventricle 68 versus 254 (P < 0.001), and ascending aorta 56 versus 185 (P < 0.001). In the CO_2_ group, the median number of detectable microemboli after CPB fell to zero 7 min after CPB versus 19 min in the control group (P < 0.001). This study design allowed us to follow the movements of the microemboli over a period of time and they behaved according to a characteristic pattern. First, an early peak occurred just after release of the aortic cross-clamp. Most of the microemboli were then whirling around in the left ventricle and the left atrium and were not propagated forward, whereas only a small fraction appeared in the ascending aorta. A second peak arose when the beating heart was being filled and started to eject blood during weaning from CPB. During this phase, most microemboli originated from the pulmonary veins. They first appeared as floating strings of pearls at the roof of the left atrium, they were then propagated forward to the left ventricle and finally ejected into the ascending aorta. Despite thorough surgical de-airing, new microemboli continued to pop up in the left atrium even up to 20 min after end of CPB. The second peak agrees with our earlier transcranial Doppler study during open-heart surgery, in which we found that most microemboli reached the brain during and after weaning from CPB [8]. This is the critical moment, as it is during weaning from CPB that the heart starts ejecting microemboli to the brain. Thus, it is then that the difference in the number of microemboli between the CO_2_-treated patients and the controls comes to the fore [8]. Furthermore, it should be kept in mind that in the CO_2_-treated patients, the microemboli were not only fewer in number but also differed from those in the untreated group as to their composition [15]. They consisted of CO_2_ and not of air.

## Conclusion

The mini diffuser was effective for carbon dioxide de-airing, i.e. < 1% remaining air **(**Table 2**)**, of a minimal invasive cardiothoracic wound cavity model with and without intermittent rough suction as well as in patients undergoing minimal invasive aortic valve surgery.

**Table 2:** Table summarizing comparison between the experimental results of the new device to the control

Insufflation device	Open-ended tube (1 mm inner diameter)		Mini-diffuser	
CO_2_ flow rate	5 L/min	10 L/min	5 L/min	10 L/min
Mean air content % in wound model	43%	49%	< 1%	< 1%

## Abbreviations

C: Celsius
CO_2_: Carbon dioxide
L/min: Liters per minute
m: Meter
mbar: millibar
mm: millimeter
mm^2^: square millimeter
O_2_: Oxygen
PVC: Poly vinyl chloride

## References

[1] Fries CC, Levowitz B, Adler S, Cook AW, Karlson KE, Dennis C (1957). Experimental cerebral air embolism. Ann Surg.

[2] Kunkler A, King H (1959). Comparison of air, oxygen and carbon dioxide embolization. Ann Surg.

[3] Eguchi S, Sakurai Y, Yamaguchi A (1963). The use of carbon dioxide gas to prevent air embolism during open heart surgery. Acta Med Biol.

[4] Goldfarb D, Bahnson HT (1963). Early and late effects on the heart of small amounts of air in the coronary circulation. J Thorac Cardiovasc Surg.

[5] Spencer FC, Rossi NP, Yu S-C, Koepke JA (1965). The significance of air embolism during cardiopulmonary bypass. J Thorac Cardiovasc Surg.

[6] Hindman BJ, Dexter F, Subieta A, Smith T, Cutkomp J (1999). Brain injury after cerebral arterial air embolism in the rabbit as determined by triphenyltetrazolium staining. Anesthesiology.

[7] Borger MA, Peniston CM, Weisel RD, Vasiliou M, Green RE, Feindel CM (2001). Neuropsychologic impairment after coronary bypass surgery: effect of gaseous microemboli during perfusionist interventions. J Thorac Cardiovasc Surg.

[8] van der Linden J, Casimir-Ahn H (1991). When do cerebral emboli appear during open heart operations? A transcranial Doppler study. Ann Thorac Surg.

[9] Tingleff J, Joyce FS, Pettersson G (1995). Intraoperative echocardiographic study of air embolism during cardiac operations. Ann Thorac Surg.

[10] Ng SW, Rosen M (1968). Carbon dioxide in the prevention of air embolism during open-heart surgery. Thorax.

[11] Eguchi S, Bosher LH (1962). Myocardial dysfunction resulting from coronary air embolism. Surgery.

[12] Moore RM, Braselton CW (1940). Injection of air and of carbon dioxide into a pulmonary vein. Ann Surg.

[13] Persson M, Svenarud P, van der Linden J (2004). What is the optimal device for carbon dioxide de-airing of the cardiothoracic wound and how should it be positioned?. J Cardiothorac Vasc Anesth.

[14] Svenarud P, Persson M, van der Linden J (2003). Efficiency of a gas-diffuser and influence of suction in carbon dioxide de-airing of a cardiothoracic wound cavity model. J Cardiothorac Vasc Anesth.

[15] Svenarud P, Persson M, van der Linden J (2004). Effect of CO2 insufflation on the number and behavior of air microemboli in open-heart surgery: a randomized clinical trial. Circulation.

